# First Annual Meeting of the Stockholders of the Ohio Dental College

**Published:** 1888-05

**Authors:** E. G. Betty

**Affiliations:** Cincinnati


### First Annual Meeting of the Stockholders of the Ohio Dental College.

Cincinnati, March 6th, 1888.

The first annual meeting of the stockholders of the Ohio College of Dental Surgery convened in the lecture room of the College at 9 o'clock, A. M., on the above date.

On motion of Dr. Leslie, Dr. G. W. Keely was elected chairman, and Dr. E. G. Betty, secretary.

On motion Dr. F. A. Hunter read the minutes of the last meeting of the College Association. Dr. Leslie offered the following resolution seconded by Dr. J. I. Taylor:

*Preamble,* Whereas on February 19th, 1852, at a meeting of the stockholders of the Ohio College of Dental Surgery, held in this city, an adjunct association was formed and named the Ohio

Dental College Association. This association devised and adopted a constitution, in which they allowed two classes of stockholders —one who had paid one hundred dollars for a share of stock of the Ohio College of Dental Surgery—another who paid annually six per cent. interest on one hundred dollars, the par value of one share.

No doubt the worthy and eminent men, the founders of this association, believed that an organization of this character was desirable and judicious in the early history of this institution, as it might secure the interest of many dentists in its behalf who may have declined to pay for a share of stock at once, and realizing the difficulties to raise funds to meet obligations, it was a wise arrangement; but, as the Ohio College of Dental Surgery is now entirely out of debt, we do not need the aid of this adjunct association, and as we are the only legal body by virtue of our corporation, and known as the Ohio College of Dental Surgery, and perfectly competent by the action of its annual meetings and duly elected Board of Trustees to do all that this adjunct, the Ohio Dental College Association can do, I move the adoption of the following resolutions:

*Resolved*, That the adjunct association known as the Ohio Dental College Association devised by the stockholders of the Ohio College of Dental Surgery be dissolved and its constitution null and void, but its records, which have been virtually the proceedings of the Ohio College of Dental Surgery, under the name of the Ohio Dental College Association, since its organization to date in all that relates to Trustees, certificates of stock and the general management of the affairs of the Ohio College of Dental Surgery, we hereby accept as binding on this body and confirm the same.

*Dr. F. A. Hunter* thought it better not to adopt the resolution at present.

*Dr. J. Taft* moved to table the resolution for one year; seconded by *Dr. F. A. Hunter.* By common consent, Dr. Leslie was permitted to make remarks upon the motion—he favored the abolishment of the Ohio Dental College Association. On vote, the resolution came up again for consideration.

*Dr. H. A. Smith* expressed a doubt of the right of a minority of the stockholders taking such action.

*Dr. J. Taft* called for a division of the resolution at the word "void," *i. e.* that part relating to the ratification of the proceedings of the Ohio Dental College Association. After some discussion, Dr. Taft withdrew his call for division.

A ballot by shares was then taken on the Preamble and resolution and it was carried unanimously—52 ballots cast.

*Dr. Leslie* submitted a schedule of rules for conduct of business—laid on the table for consent.

Adjourned till 9 A. M., March 7th.

---

March, 7, 1888—9.30 A. M.

The Stockholders re-assembled at this hour. Dr. Keely in the chair. The minutes of the previous session were read and approved. Dr. Leslie's rules of conduct of business were brought up for action and considered by sections, and were adopted singly and then as a whole. They are as follows:

### RULES FOR THE CONDUCT OF BUSINESS DURING THE ANNUAL MEETINGS OF THE STOCKHOLDERS OF THE OHIO COLLEGE OF DENTAL SURGERY.

SECTION I. That the annual meeting of its stockholders be held on the Tuesday preceding the first Wednesday in March, in the college building at 10 o'clock, A. M. A majority of stockholders present at this meeting authorized to begin and transact business by electing a president and secretary for the meeting, provided a majority of the legal stock is represented.

SEC. II. The minutes of the previous meeting to be read.

SEC. III. That no person is a stockholder of the Ohio College of Dental Surgery who does not hold a certificate for his stock, or whose name is not on its recorded list of stockholders; but if any person claiming to be a stockholder, who may have lost or mislaid his certificate, his claim shall be referred to the Board of Trustees for their decision.

SEC. IV. That each stockholder has the right to as many votes as he owns shares of stock, at all meetings of the same; and when a stockholder desires to vote by proxy—the person casting said vote, shall submit to the secretary of the meeting the stockholder's written authority for the same.

SEC. V. The President of the Board of Trustees will make a written report of the general condition of college affairs; said board being authorized to issue diplomas, appoint or remove professors, and do whatever may be necessary as our legal representatives.

SEC. VI. The Dean of the Faculty will make a written report respecting the college department during the college session.

SEC. VII. The meeting shall elect *three trustees* to take the places of those who had been elected by the Ohio Dental College Association, whose term now expires. The members of the whole board to be nine (9); and said board will elect its own president and secretary.

SEC. VIII. That the secretary of the Board of Trustees obtain and insert in his record book a perfected list of recognized stockholders.

On motion the schedule as a whole was adopted.

At 12.30 P. M. March 7th, 1888, in adjourned meeting, Drs. C. R. Taft, Jas. Leslie and G. W. Keeley were re-elected trustees.

Adjourned till 9 A. M., March 8th.

---

March 8, 1888—9.30 A. M.

The stockholders re-assembled. Dr. Keely in the chair. Minutes of the previous meeting read and approved. On motion of Dr. Taylor the minute book of the defunct College Association was adopted as the official record book of the stockholders. Dr. Leslie, the treasurer of the Board of Trustees made a report showing about $100.00 in the treasury. Dr. G. W. Keely then made a statement showing the condition of the college; after which the stockholders adjourned till the next annual meeting.

E. G. BETTY, SECRETARY.

